# Patient preferences for direct-to-consumer telemedicine services: a nationwide survey

**DOI:** 10.1186/s12913-017-2744-8

**Published:** 2017-11-28

**Authors:** Brandon M. Welch, Jillian Harvey, Nathaniel S. O’Connell, James T. McElligott

**Affiliations:** 1 Department of Public Health Sciences, 135 Cannon St., 405L, Charleston, SC 29455 USA; 2 Department of Healthcare Leadership & Management, 151-B Rutledge Avenue, MSC 962, Charleston, SC 29425 USA; 3 Department of Public Health Sciences, 135 Cannon St., 300, Charleston, SC 29455 USA; 4 Department of Pediatrics, 135 Rutledge Avenue, Charleston, SC 29425 USA

**Keywords:** Direct-to-consumer telemedicine, Telehealth, Home telecare, Remote consultation

## Abstract

**Background:**

Direct-to-consumer (DTC) telemedicine providers has the potential to change the traditional patient-physician relationship. Professional medical organizations recommend that telemedicine exist within the medical home. This study aims to understand patients’ preferences and desires for DTC telemedicine.

**Methods:**

We conducted a nationwide survey of 4345 survey respondents demographically balanced to represent the United States adult population. The survey consisted of questions assessing the respondents’ attributes and their willingness and comfortability using telemedicine as well as the importance and desired attributes of a provider providing care via telemedicine.

**Results:**

Relatively few respondents (3.5%) had ever had an online video visit with their care provider. Respondents were more willing to see their own provider via telemedicine than unwilling (52% vs. 25%). Additionally, respondents were less willing to use telemedicine to see a different provider from the same healthcare organization (35%) and were least willing to see a different provider from a different organization (19%). Forty-one percent of respondents felt it was unimportant that their current provider offer telemedicine, and only 15% would consider leaving their current provider to a new provider who offers telemedicine as an option. More than half (56%) of respondents felt it was important to have an established relationship with a provider they’re having a telemedicine visit with. Nearly two-thirds of respondents (60%) felt it was important for a telemedicine provider to have access to their health records.

**Conclusions:**

Patients prefer to use telemedicine with their own doctor with whom they have an established relationship.

## Background

Synchronous, real-time telemedicine is quickly establishing a place in routine clinical care as an accessible, efficient care that can reduce costs while maintaining quality of care and patient satisfaction [1, 2]. Real-time telemedicine can be delivered between two clinic sites (e.g., hospital to remote hospital/clinic) or directly between a care provider and the patient at home or work. Direct-to-consumer (DTC) telemedicine can provide immediate and convenient access to a care provider that usually costs less than a standard in-person urgent care or emergency room visit [3]. DTC telemedicine has also been shown to increase access to care to those without a primary care provider [4, 5].

DTC telemedicine can be delivered through three primary approaches. First, DTC telemedicine can be provided to patients by their care provider with whom they have an established, traditional doctor-patient relationship. With this approach, DTC telemedicine complements the in-person visits by providing an option for patients to see the provider in person or by telemedicine, depending on the situation and context of the visit. This approach offers convenience to patients while providing continuity of care with the same provider who sees patients in person. The second DTC telemedicine approach entails patients seeing a different care provider from the same organization as the primary care provider. Being at the same organization, this care provider can view and update patients’ health records, provide a level of care within the patients’ medical home, see them, and update health records with notes from the telemedicine encounter. This approach maintains a connection to patients’ established medical home and provides immediate, on-demand questions and care during regular as well as off hours. The third and final DTC telemedicine approach is provided by a care provider from an organization outside the patients’ medical home, where there is little or no previous doctor-patient relationship or coordination with the patients’ primary care provider. New DTC telemedicine companies and others are examples of this approach [5]. DTC telemedicine companies usually either have contracts with insurance companies and healthcare organizations or charge patients directly for their services. The advantage of this approach is that these DTC services are available to anyone by contacting one of these companies; despite DTC medicine’s ease of use, many traditional care providers and organizations have yet to utilize it with their patients.

In response to the growing utilization of DTC telemedicine and the rise of DTC companies, the American College of Physicians released a position statement in 2015 which it stated its support of telemedicine within the context of an established patient-provider relationship in a medical home if it meets the same standards of practice as in-person care [6]. In support of these standards and expectations, some state medical boards require an in-person exam before a telemedicine consult can take place [7].

With DTC telemedicine companies pushing for telemedicine outside the traditional medical home and the professional organizations supporting the expansion of DTC telemedicine within the patients’ medical home, little is known about patient preferences for DTC telemedicine. Several reports indicate that a majority of patients would be comfortable with telemedicine; however, these studies often fail to differentiate between DTC telemedicine delivered by patients’ own care provider (within the medical home) versus a telemedicine visit with a provider with whom they have little to no prior relationship (outside the medical home) [8–11]. The purpose of this study is to understand patients’ potential use and preferences for DTC telemedicine with regards to provider context.

## Methods

A nationwide survey using SurveyMonkey Audience (Palo Alto, CA) was conducted to glean a balanced, nationwide representation of the adult U.S. population, which was weighed by gender, race, and education. SurveyMonkey Audience, commonly used in academic research, has participants complete a demographic profile that includes age, sex, education level, geographic location, socioeconomic status, and other relevant demographic information [12, 13]. This demographic information is then used to randomly recruit a population balanced to correlate with the general U.S. population. To maintain the integrity of response data, SurveyMonkey Audience limits the number of surveys a member can participate in per week, rewards members with non-cash incentives (charitable donations and sweepstakes entries), and regularly benchmarks the members [14].

Survey questions were derived from prior surveys used in health services research and consumer use of telemedicine [8–11, 15]. The survey expanded on the prior surveys by asking respondents about telemedicine under three different scenarios: (1) with their current primary care provider, (2) with a different provider from the same healthcare organization as primary care provider, and (3) with a different provider from a different healthcare organization. The survey was reviewed and revised among approximately ten telehealth and health policy experts at the Medical University of South Carolina. The reviewers provided feedback related to the clarity of the questions, logical flow of the survey, length of time required to complete the survey, as well as appropriateness of survey content and response choices. The final survey instrument consisted of 17 questions. Six of these assessed the respondent’s health status, insurance type, and relationship with primary care provider, as well as whether the provider offers telemedicine video visits, and the respondent has had a telemedicine visit with his/her provider. Questions were included to assess both a respondent’s willingness to use telehealth and comfort with using telehealth technology. The survey used two 5-point Likert scale matrices to represent a respondent’s willingness and comfortability. Finally, four Likert scale questions assessed the importance and desired attributes of a provider practicing telemedicine. Demographic information used in our analysis was collected by SurveyMonkey prior to our study. (See Appendix A for a copy of our survey.) At the beginning of the survey all participants consented to research to take part in the study, which was approved by the Institutional Review Board (IRB) at Medical University of South Carolina.

Survey data were analyzed using R 3.1.1 and SAS 9.4 (SAS Institute, Cary, NC) [16]. Fully conditional specification multiple imputation was used to impute missing data for the covariates of interest used in statistical models in SAS 9.4, and analysis was performed on complete survey responses [17]. To better represent the U.S. population, survey data were weighted (with upper-bound truncated at 3.5 per subgroup) to match the gender, race, and education of the 2013 U.S. population according to the U.S. Census Bureau. To assess differences between willingness/comfort scenarios, while adjusting for potential confounders, we dichotomized the outcome to “willing” (with choices “very willing,” “willing,” and “neutral”) vs. “unwilling” (with choices “unwilling” and “very unwilling”). “Neutral” was grouped with “very willing” and “willing” because we believed these individuals would be more likely to use rather than not use telemedicine. A model was fit using a logistic link via a generalized estimating equation which adjusts for age, gender, education, race, income, geographic region, and insurance type. A weighted Cohen’s kappa assuming Fleiss-Cohen weights was used to assess inter rater agreement between questions of comfort/willingness across the question scenarios. Differences in proportions were tested using a score test.

## Results

Between October 22 and 30, 2015, 4438 survey participants were randomly selected by SurveyMonkey to participate. The majority participated —4345 or 97.9%. From the unweighted sample of these respondents, all answered the primary questions of interest. Approximately 4% of each covariate were missing responses, with the exception of education level with 1145 missing responses (26.4%). All respondents selected a response for income, although 628 selected “prefer not to answer” (14.5%), a response that was treated as a level of response regarding income levels—rather than treated the response as missing.

From the weighted sample, 54% of respondents were female, 78% were white, 67% had some college level education or graduate level education. Forty-six percent of respondents of respondents had a household income of less than $50,000 per year, 84% of respondents reported being in ‘good’ health or better, and 48% reported having private health insurance. Eighty-four percent of the respondents reported having a current primary health care provider, with 39% going to their current provider for more than 5 years. Of those who had a primary care provider, 50% had seen their provider once or twice in the past year. (See Table 1 for full results).

**Table 1:** Demographics of Respondents

Gender			
female	54%		
Male	46%		
Age		Education	
18–29	22%	HS or Less	33%
30–59	52%	College	55%
60+	26%	Graduate	12%
Race		lncome	
White	78%	No Response	14%
Hispanic/Latino	8%	<50$	46%
Black	7%	$50 -$100 K	22%
Other	8%	>$100	18%
Health		Insurance type	
Excellent	14%	Private/Other	58%
Very Good	35%	No Insurance	11%
Good	36%	Medicare	17%
Fair	13%	Medicaid	7%
Poor	3%	Other Gov	7%

(Weighting to US population; *n* = 4345)

### Use of telemedicine with current primary care provider

Only 5.3% of respondents that have a primary care provider reported knowing that their primary care provider offers online video visit, with 31% unsure. Only 3.5% of respondents had ever used online video visit to meet with their provider. Furthermore, of those without a primary care provider, only 4.6% reported ever using telemedicine. These results are consistent with industry reported rates [18–22]. There is not a significant difference in telemedicine utilization (3.5% vs. 4.6%; chi-square > 0.05) between those with a primary care provider and those without.

### Willingness to use telemedicine

**Fig. 1:**
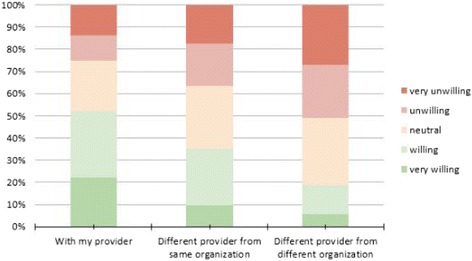
Willingness to use telemedicine

More than twice as many respondents were willing to see their own provider via telemedicine than were unwilling. Over half (51.9%) of respondents would be willing (22.1% very willing and 29.8% willing) to see their own provider by telemedicine. Conversely, one quarter (25.3%) of respondents would be unwilling to see own provider. Nearly 22.7% were neither willing nor unwilling. With respect to seeing a different provider from the same healthcare organization by telemedicine, 34.9% of respondents are willing or very willing, whereas 36.7% would be unwilling. Finally, respondents were least willing to see a different provider from a different organization by telemedicine, with only 18.6% of respondents being willing (5.8% very willing and 13% willing) and 51% unwilling (27% very unwilling, 24% unwilling, *p* < 0.001). See Fig. 1.

Using GEE models, we found statistically significant decreased odds of willingness as respondents became further detached from their own provider. The estimated within-respondent correlation across the three question scenarios was 0.60, suggesting that an individual’s willingness under one condition is moderately to strongly correlated with their willingness under the other two conditions. While over 50% of respondents are either very willing or willing to use telemedicine when it is with their own provider, only about one-third are when it is with a different provider at their same institution, and under 20% are when it is with a different provider at a different institution. In summary, respondents become less willing to use telemedicine as they become further detached from their own provider.

### Comfort using telemedicine

**Fig. 2:**
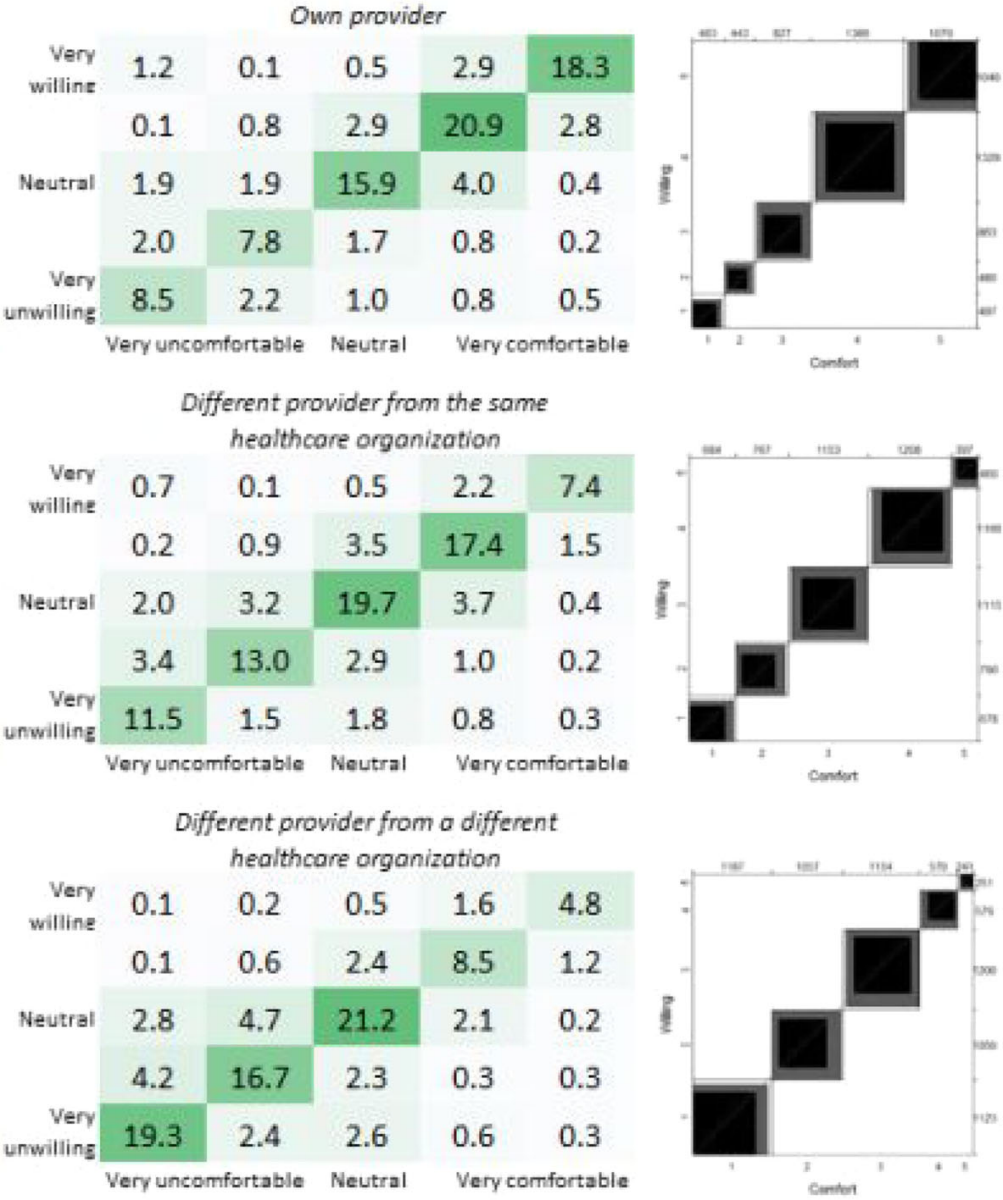
Percentage of respondents’ willingness and comfort to have online video visits

Results for “comfort” correlated closely with results for “willing.”—with 53.7% comfortable and 24.5% uncomfortable seeing own provider by telemedicine, 35.0% comfortable and 36.3% uncomfortable seeing a different provider same organization by telemedicine, and 18.6% comfortable and 52.7% uncomfortable seeing different provider from a different organization by telemedicine. These results show a very strong agreement within respondents’ willingness and comfort to use telemedicine under each given provider scenario with weighted kappa estimates of 0.80, 0.78, and 0.79. See Fig. 2. For a matrix matching the response to each question and percentage of results for each respondent.

### Desired attributes of telemedicine provider

Forty-one percent of respondents did not feel that it was important that their current provider to offer telemedicine, whereas 19.8% of respondents felt it was. Overall, 14.9% would consider switching to a new provider who offers telemedicine as an option, whereas 55.9% would not consider switching providers (*p* < 0.001). Of those who felt it was important that their current provider offer telemedicine, 41.7% would consider switching, whereas of those who said it was unimportant, only 15.6% would (*p* < 0.001). The more important respondents consider telemedicine, the more likely they are to a provider that offers it (Pearson correlation = 0.49, <0.001). Having an established relationship with a provider with whom they are having a visit was found to be important, with 56.3% agreeing and only 19.3% disagreeing (*p* < 0.001). Two-thirds of respondents (60.3%) believe it is important for a telemedicine provider to have access to their health records, while 19% do not.

## Discussion

The goal of this survey was to better understand patient preferences for different care models of DTC telemedicine. To our knowledge, this is the first study to assess consumers’ desire and willingness to use DTC telemedicine with providers whom they have an established relationship, compared to providers whom they do not. Respondents feel that it is important to have an established relationship with a telemedicine provider and are less willing to see a provider with whom they are unfamiliar. Further investigation is needed to understand how consistent these results are with patient preferences for non-telemedicine care. Regardless, our results show patients are more willing and comfortable to receive DTC telemedicine care by a provider with whom they have a relationship.

There are several implications of these findings on the future growth of DTC telemedicine. First, there appears to be relatively small patient demand for care providers to offer DTC telemedicine in general. Relatively few patients knew whether their provider offered telemedicine, and twice as many respondents felt it was unimportant for their care provider to offer telemedicine. While the telemedicine market is growing, healthcare will likely not see significant changes to its delivery landscape until telemedicine grows in demand among patients.

Second, due to patient preferences, it is plausible that DTC telemedicine offered by traditional care providers will have higher utilization rates compared to DTC telemedicine companies, which utilize a pool of providers. We expect to see the greatest growth of telemedicine within the context of patients’ medical home, the preferred model of coordinated primary care in this country [23, 24]. Importantly, these findings support an advocacy for payers and employers to include telemedicine offerings that work with local healthcare organizations to offer telemedicine to their patient population within the context of the medical home.

### Limitations

This study has several limitations to consider. First, the respondents were recruited through the online survey platform SurveyMonkey Audience. This sample may produce a bias towards individuals more likely to be online (more educated, wealthy, white, etc.). However, this population also represents the segment of the population that would also be most likely to participate in an online telemedicine call, likely skewing results more in favor of DTC telemedicine. Additionally, because we weighted results by gender, race, and education based on a recent U.S. current population survey, we alleviated some of this bias. Also, extreme weights for underrepresented subgroups were truncated to 3.5, limiting the degree to which they were up-weighted. A sensitivity analysis was performed to see how statistical inference changed between unweighted and weighted data; for the formal statistical analyses presented, there was little to no differences in statistical inference made in this manuscript. Second, the survey methodology is not as rigorous as other techniques such as direct, structured interviews. In addition, it included a small number of survey questions, limiting the ability to achieve internal validity. However, this survey is not intended to be a comprehensive assessment of patient preferences; rather, it is a first step in understanding patient preferences for telemedicine based upon relationship with the provider. Third, the telemedicine delivery model assessed in this study focuses on patient-to-provider, or DTC telemedicine. Other telemedicine models that have higher adoption rates (e.g., hub-and-spoke specialty care) were not considered as part of this study, so our results do not represent all of telemedicine. Finally, the results are based on patients’ self-reported responses to hypothetical situations. Patient preferences and use of telemedicine may yield different results in real situations.

### Future direction

We are planning to further explore trends among subpopulations of survey respondents to understand how different patient segment affect responses. There are likely many interesting trends related to age, insurance type, race, gender, education, income, and health status that impact patients’ telemedicine preferences. Additionally, this survey represents only a single point in time (Oct. 2015) and does not assess trends or changes in patient demand over time. Therefore, we will conduct periodic assessments in the future to determine trends among patients for telemedicine over time. There is a small percentage of patients who are comfortable with a telehealth provider they have not met with in person. We would like to further examine the priorities and needs of these individuals as well as the implications of and the long-term impact of their use of healthcare outside a traditional medical home. We also plan to explore respondents’ willingness and comfort in seeing a different provider under various scenarios, such as in an urgent situation or when their provider is unavailable. Finally, the survey sample is from the US population. While we expect trends to be similar in other western countries, it would be interesting to explore interest in DTC telemedicine in various countries around the world.

## Conclusion

Patients prefer to use DTC telemedicine with their own doctor with whom they have an established relationship. This preference corresponds to the recommendations by American College of Physicians that supports telemedicine within the context of the medical home. These results encourage the advocacy for the adoption of DTC telemedicine by local, traditional care providers has an element of their continuum of care for the populations served.

## Abbreviation

DTC: Direct-to-consumer
